# A large and distinct skin impression on the cast of a sauropod dinosaur footprint from Early Cretaceous floodplain deposits, Korea

**DOI:** 10.1038/s41598-017-16576-y

**Published:** 2017-11-27

**Authors:** In Sung Paik, Hyun Joo Kim, Hoil Lee, Seongyeong Kim

**Affiliations:** 1 Department of Earth and Environmental Sciences, Pukyong National University, Busan, 48513 Republic of Korea; 2 Korea Institute of Geoscience and Mineral Resources, Daejeon, 34132 Republic of Korea; 3 School of Earth and Environmental Sciences, Seoul National University, Seoul, 08826 Republic of Korea

## Abstract

The occurrence and features of skin impressions in a sauropod footprint, the largest (>50 cm in diameter) reported to date for this taxon, from the Lower Cretaceous Haman Formation (Albian) in Korea are described, and its preservation and paleoenvironmental implications are interpreted. The skin impression-bearing deposits are floodplain sediments formed by sheetflood processes. The large impression is preserved in silty mudstone with microbial lenses and wisps overlying a planar- to cross-laminated and fine-grained sandstone to siltstone bed. The paleoenvironment of the skin impression-bearing deposits is interpreted as a saline sandflat to mudflat where microbial mats can form around lakes or ponds under semi-arid paleoclimatic conditions with alternating wetting and drying intervals. These paleoenvironmental conditions would have permitted the distinct preservation of skin impressions in a dinosaur footprint. The observations here suggest that some sauropod dinosaurs in the Cretaceous had a well-developed polygonal skin texture covering nearly the whole of their foot pads, as seen in modern elephants, which would increase stability when walking on muddy and wet ground.

## Introduction

Although several distinct skin impressions in Mesozoic reptile footprints have been documented^1–4^, skin impressions in dinosaur footprints are very rare compared to the large number of dinosaur tracks discovered around the world^5,6^. Skin impressions in dinosaur footprints have been documented from only a few isolated casts of footprints^7–11^ and tracks^12,13^. The Cretaceous fluvio-lacustrine deposits in Korea contain innumerable dinosaur tracks^14^. However, skin impressions in dinosaur footprints have very rarely been observed in these tracks^15,16^. Although several casts of dinosaur footprints are preserved in the Cretaceous deposits of China^17,18^, few skin impressions have been observed. Here, we report a large and distinctly preserved skin impression on the cast of a foot pad print of a sauropod dinosaur. We also describe some diffuse and partial skin impressions in other dinosaur footprints. These findings provide insight into the unique conditions for the preservation of skin impressions in dinosaur footprints and the paleoenvironmental and paleoecological implications of these insights.

Dinosaurs had foot pads, but it is unclear why skin impressions in dinosaur footprints are extremely rare. Some studies have suggested that the predominance of dinosaur tracks as underprints or overtracks, the inadequate preservation conditions, or the erosion might explain the rare occurrence of these skin impressions^11^. However, the common presence of distinctly preserved dinosaur footprints without skin impressions and the very limited number of impressions on numerous casts of dinosaur footprints suggest that specific preservation conditions may have limited the occurrence of footprints with skin impressions.

**Figure 1:**
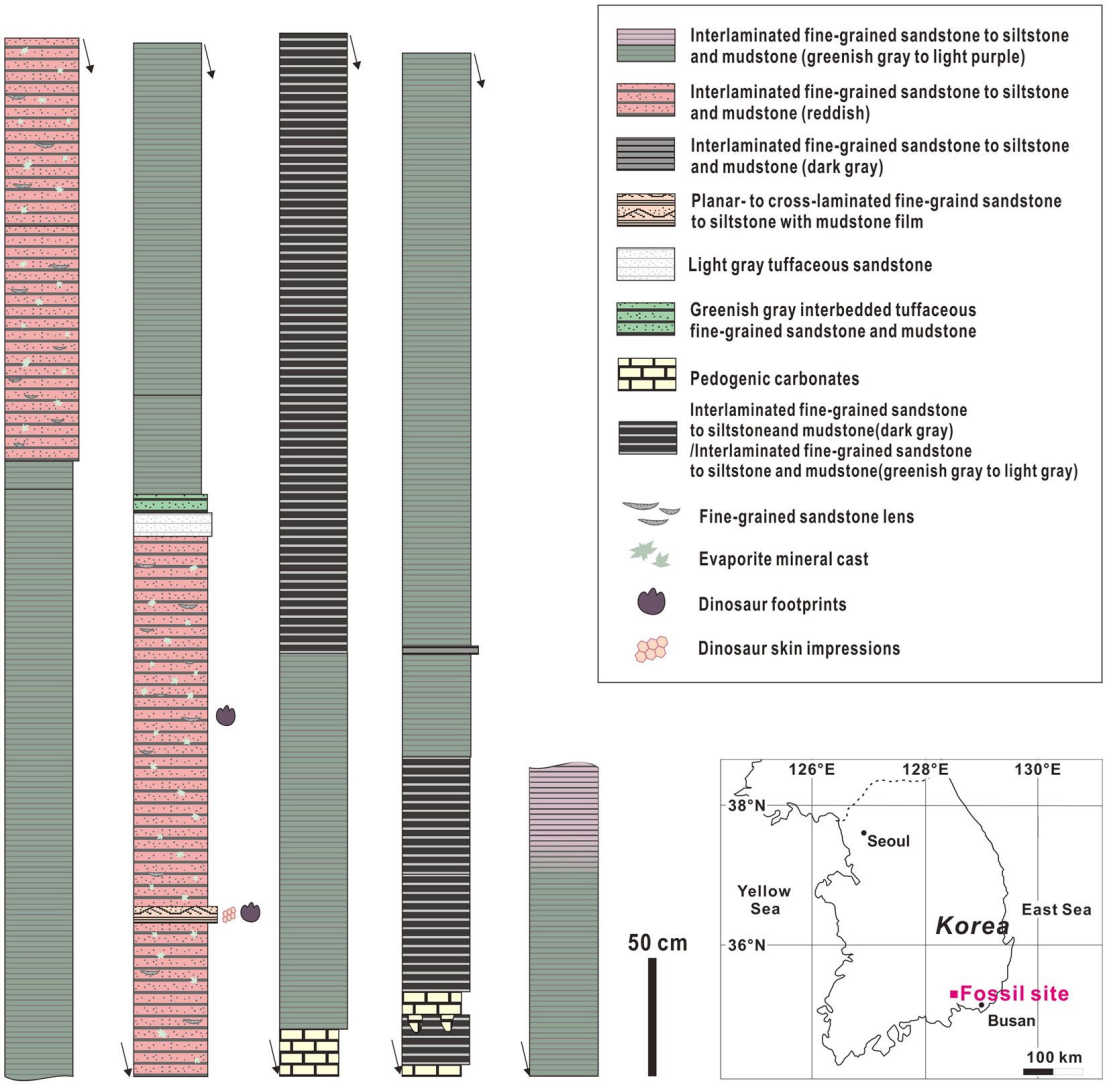
Geographical location and stratigraphic sections of the dinosaur skin impression-bearing deposits. The inset was drawn using CorelDRAWX5 (www.coreldraw.com).

**Figure 2:**
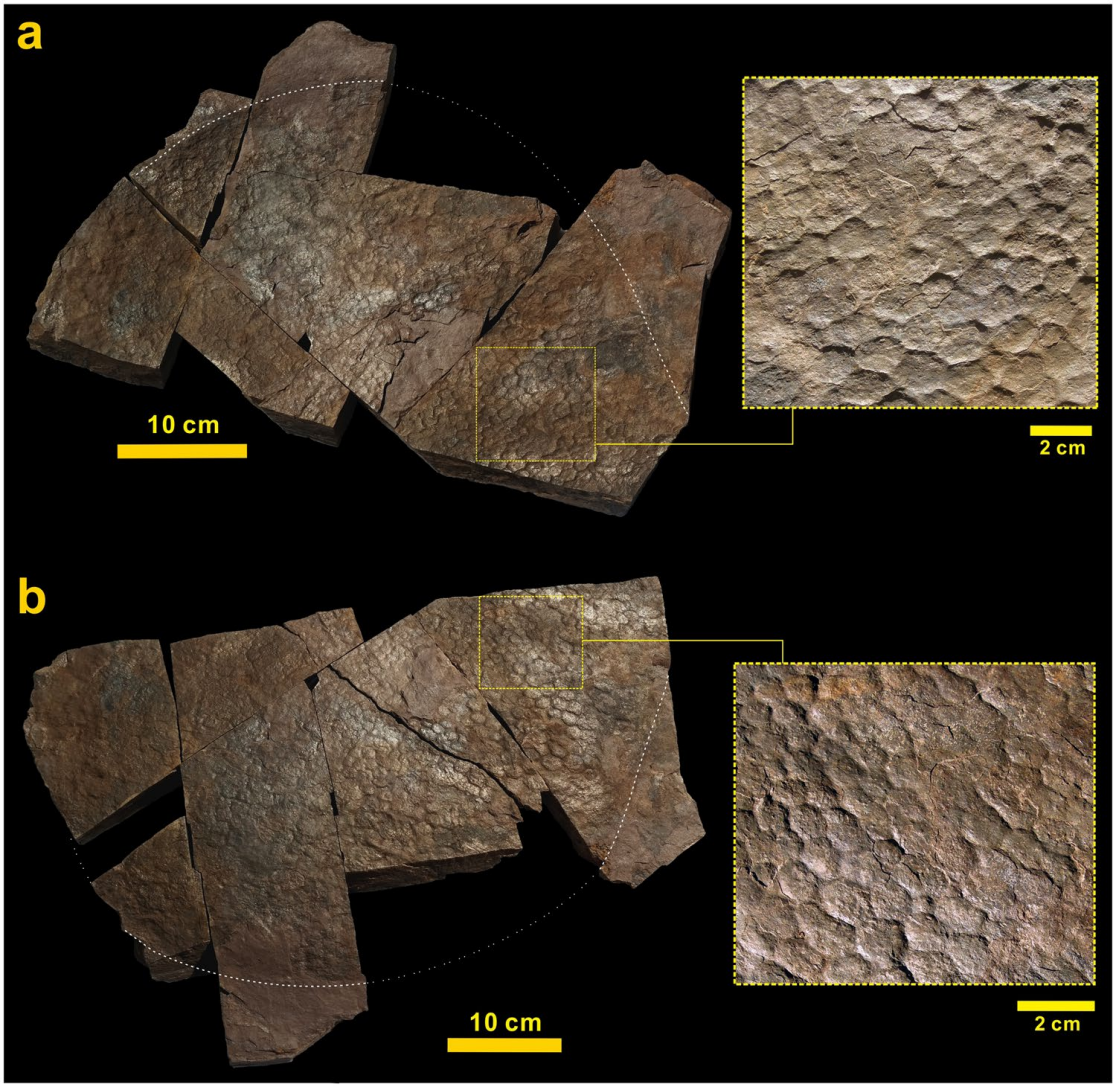
Distinct skin impressions in a sauropod footprint (**a**) and on its cast (**b**) described in this study.

**Figure 3:**
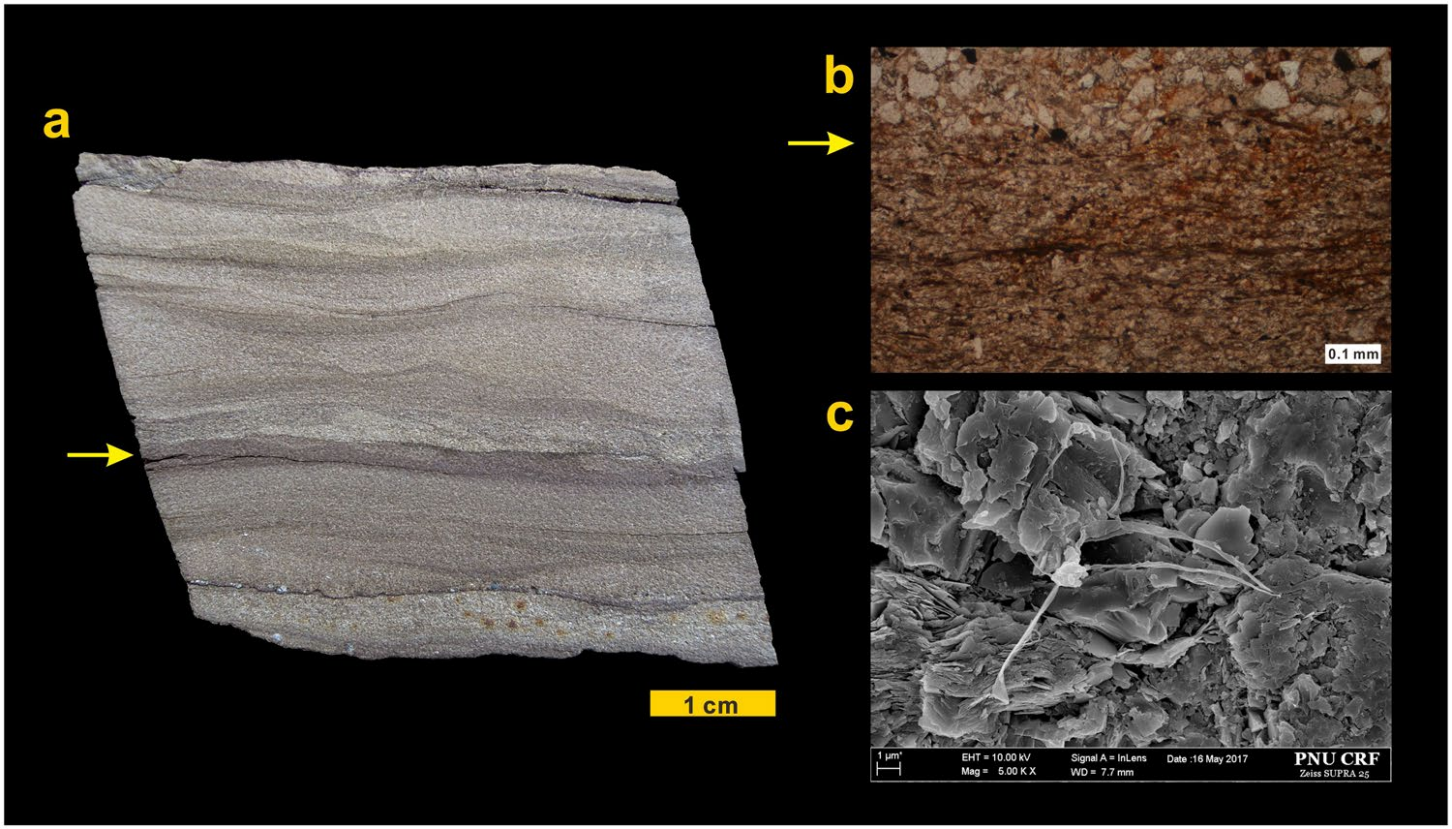
Features of the dinosaur skin impression-bearing bed. (**a)** Sectional view showing the skin impression-bearing mudstone lamina (arrow) between planar- to cross-laminated fine-grained sandstone beds. (**b**) Thin section photomicrograph of the skin impression-bearing mudstone (arrow) with thin wisps of organic material. (**c**) FE-SEM photographs of a thin section of the skin impression-bearing mudstone showing the presence of filament-like microbes bound in clay minerals.

Here, an unusually large (>50 cm in diameter) distinct skin impression in a sauropod footprint is documented in Early Cretaceous floodplain deposits (Haman Formation) exposed in Gunbook-myeon, Haman-gun, Gyeongsangnam-do, Korea (Figs 1and 2). A diffuse skin impression in another sauropod footprint (Fig. 3) and partially preserved skin impressions in patches in another dinosaur footprint (Supplementary Information Fig. 1) were also observed in these deposits. These observations reveal a spectrum of preservational styles: impressions in nearly the entire area of a footprint, impressions in only part of a footprint, and distinct to diffuse impressions showing the polygonal skin texture of the foot in a footprint. The skin impressions observed in this study may provide insights into the preservation conditions of dinosaur footprint skin impressions and the reasons for their rarity despite the occurrence of numerous dinosaur tracks around the world.

## Results

### Skin impression-bearing deposits

The skin impression-bearing sediments are floodplain deposits formed by sheetflood processes. The deposits consist of interlaminated to interbedded fine-grained sandstone to siltstone and mudstone, planar to cross-laminated fine-grained sandstone, and calcareous silty mudstone (Fig. 1). Current and wave ripple marks and polygonal mud cracks are common in these beds, indicating that these deposits were formed on lake margins. These deposits are usually purple in colour, with the preservation of calcic and vertic paleosols, suggesting that deposition occurred during alternating wet and dry intervals and semi-arid paleoclimatic conditions. Sauropod, ornithopod, and theropod dinosaur footprints are observed intermittently in these deposits, and bird footprints in some areas.

### Skin impressions

In the purple-coloured planar to cross-laminated fine-grained sandstone beds with mudstone films, a large skin impression and its cast, with a diameter of approximately 50 cm and a distinct polygonal texture, are preserved in a subcircular and very shallow (approximately 1 cm deep) footprint of a sauropod dinosaur (Fig. 2). The specimen was found in float with several blocks produced during building  construction. The impression and its cast were revealed by parting the impression-bearing bedding plane. The impression is preserved in silty mudstone lamina (a few mm thick) overlying a planar- to cross-laminated and fine-grained sandstone to siltstone bed. The impression-bearing mudstone is composed of mostly clay minerals and some quartz silts (Fig. 3). Thin lenses and wisps of organic material are present in the mudstone lamina (Fig. 3, Supplementary Information Fig. 2). Casts of sulfate evaporite minerals are present in places in the underlying and overlying deposits, indicating deposition under semi-arid paleoclimatic conditions.

The cast of the skin impression is composed of raised hexagonal polygons ranging from 6 mm to 18 mm in width. In general, the sizes of the polygons decrease radially. The polygonal profile has a distinct V-shape with a depth < 1 mm (usually 0.5–0.8 mm). The raised hexagonal surfaces are generally arranged in rosettes, where any one polygon is in contact with five to seven other polygons. In general, the distinctiveness decreases as the impression-bearing surface becomes flatter.

**Figure 4:**
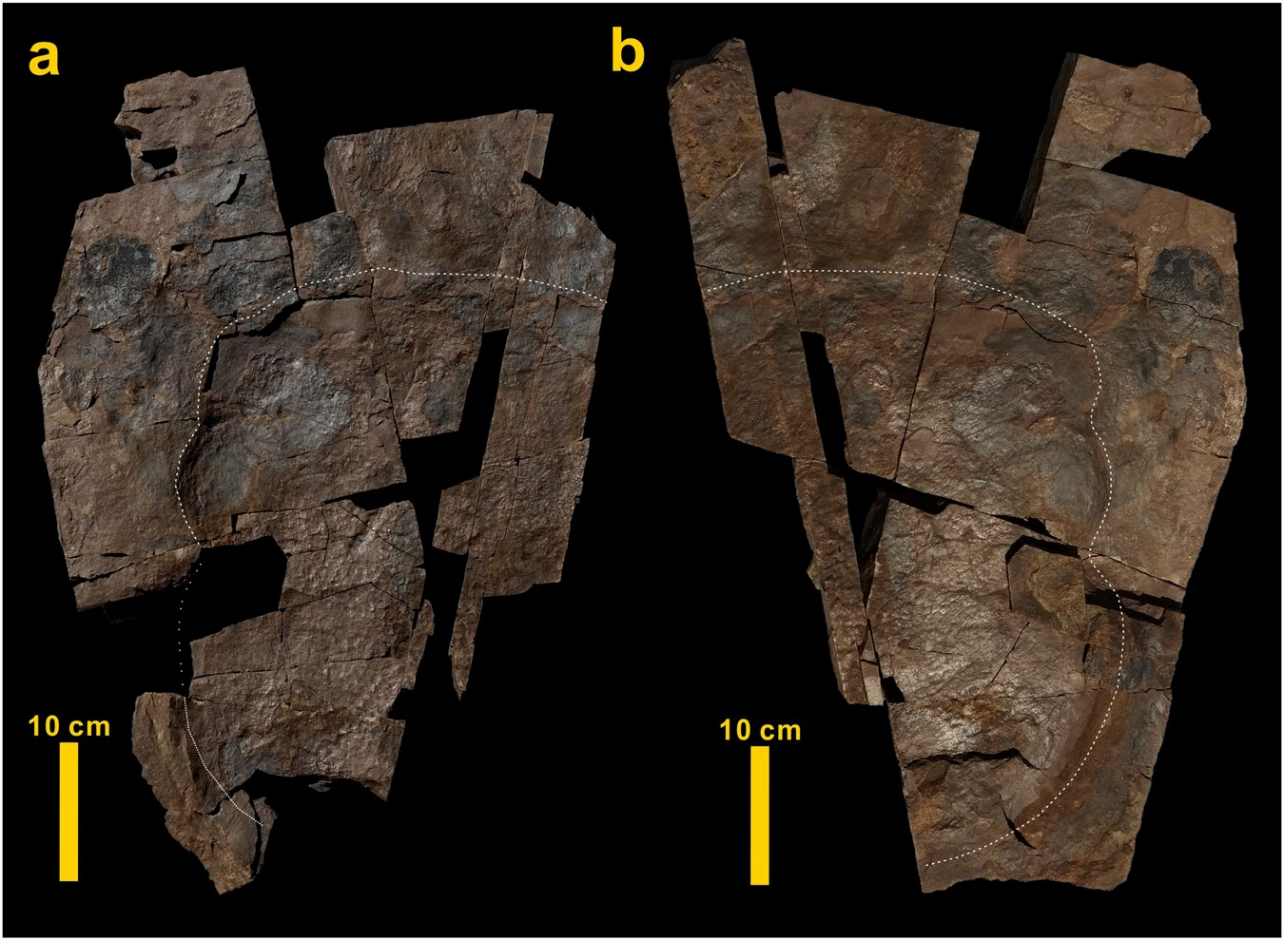
Diffuse skin impressions in a sauropod footprint (**a**) and on its cast (**b**) described in this study.

A polygonal skin impression with rather diffuse edges is also preserved in another subcircular footprint with a very shallow depression (Fig. 4). Partial and indistinct impressions of polygonal skin textures varying from diffuse polygons to ghosts of polygons have been observed in other blocks from these deposits.

The partial preservation of polygonal skin impressions was also observed in another shallow footprint in the interlaminated fine-grained sandstone to siltstone and mudstone bed (Supplementary Information Fig. 1). While the skin impression described above is preserved in a nearly complete footprint, the impressions in this footprint are partially preserved in small patches. The impression-bearing footprint is approximately 63 cm in length and 46 cm in width. The patches of skin impressions are approximately 14–28 cm in diameter, and the polygons in the impressions are approximately 7–13 mm in diameter.

## Discussion

Such variations in the size and distinctiveness of dinosaur footprint skin impressions suggest that certain conditions may be related to variations in preservation. The absence of skin impressions in many distinct dinosaur footprints and their casts suggests that the occurrence of footprints as underprints or overtracks is not the only reason for the very limited presence of skin impressions.

Some conditions may be related to the very rare occurrence of skin impressions in dinosaur footprints. First, if most dinosaurs did not have polygonal skin texture in the foot pads, as seen in most mammals, such skin impressions would have scarcely been formed in their footprints. However, the partial preservation of polygonal skin impressions in dinosaur footprints suggests that the very limited presence of such impressions is attributable to other conditions, such as the substrate on which dinosaurs walked and the taphonomic conditions after the footprints were formed.

It may be necessary for the mud substrate to have a quasi-solid state for footprints to be imprinted^5,19,20^. Thus, skin impressions of foot pads can be formed in the footprints, with slickensides formed at the margins of the footprints. The very rare association of slickensides with distinct dinosaur footprints suggests that many footprints were made when the substrate was saturated with water or that the footprints were flooded shortly after formation and covered with water for some period such that the impressions and slickensides were obliterated by water saturation. Even in cases where slickensides are closely associated with dinosaur footprints, the occurrence of skin impressions is very limited^21^. Therefore, mud surface bearing footprints with skin impressions would have been dry and semi-consolidated enough for footprint-bearing mud not to be saturated by subsequent flooding.

Consequently, the prerequisites for the preservation of distinct skin impressions in footprints can be assumed to be as follows. First, the interlaminated to interbedded nature of sand with mud drapes with thicknesses of a few millimetres would enable tracks to be impressed on the substrates. The mud drapes provided a suitable medium for the preservation of the tracks, with the underlying sands underpinning the impressions and thus preventing their disintegration^20^.

Second, the thickness and viscosity of mud must be suitable for the distinct imprinting of the foot pad skin texture. If the substrate mud is too thick or too thin, distinct skin impressions may not be imprinted. If the water-saturated mud layer is too thick, distinct skin impressions may not be formed due to the irregular deformation of the mud by trampling. If the mud layer is thinner than the thickness of the skin texture, distinct skin impressions cannot be made in the mud. Therefore, mud drapes a few millimetres in thickness would be adequate for the formation of distinct skin impressions.

There have been no reports of skin impressions with shrinkage cracks in dinosaur footprints. This suggests that the skin-imprinted surface may have been cemented early enough to prevent the formation of desiccation cracks. The formation of microbial biofilms in the trampled layer could have played a role in initial stabilization^22–24^. The observation of modern tidal-flats indicates that moist to water-undersaturated deposits with microbial mats provide the best media for the preservation of human footprints^23,25^, supporting the preservation conditions for the distinct skin impressions in dinosaur footprints postulated above.

The variations in the distinctiveness, size, and completeness of the skin impressions in dinosaur footprints observed in this study are likely related to walking speed and the roughness of the surface; dinosaurs walking more slowly on less rough surfaces would have produced more distinct footprints with larger skin impressions. Therefore, the large and distinct skin impression observed in this study could have been formed when a sauropod walked very slowly or stopped momentarily on an organic material-bound smooth surface.

Another feature of the distinct and large skin impression described here is its preservation in a very shallow and gentle depression of a footprint. These observations indicate that the substrate was hardened to an extent, enabling the substrate to resist trampling by the dinosaur. The interbedded sand and mud of the substrate on which the dinosaur walked could have rendered this medium more suitable for these conditions.

The preservation process of the distinct and large skin impression in the dinosaur footprint observed in this study can be postulated as follows. One day, a sauropod walked very slowly or stopped on a smooth and microbially active muddy surface a few millimetres thick overlying sand, forming a distinct imprint of the foot pad skin texture in the footprint. The skin impression-bearing surface dried out during the succeeding drought season, resulting in semi-consolidation of the skin impression. The footprint with the skin impression was flooded during the subsequent rainy season, burying the footprint and preserving the skin impression by sediment deposition.

The paleoenvironment of the skin impression-bearing deposits described above is interpreted as a saline sandflat to mudflat where microbial deposits can form around lakes or ponds under semi-arid paleoclimatic conditions with alternating wet and dry periods. These paleoenvironmental conditions would have permitted the distinct preservation of the skin impression in the dinosaur footprint. Similar paleoenvironmental conditions have been documented in many dinosaur track-bearing deposits around the world^6,22^. Distinct skin impressions in footprints, however, are extremely rare in these deposits, suggesting that most dinosaurs did not have a polygonal skin texture on their foot pads.

Although polygonal skin impressions have been documented from the Triassic to the Cretaceous, most have been found in Middle Jurassic to Cretaceous deposits (Supplementary Information Table S1). Although the statistical significance of these data is low, it is likely that the development of a polygonal skin texture on the foot pads represented an evolutionary adaptation for dinosaurs in the late Mesozoic. The polygonal skin texture on the foot pads of dinosaurs could have played a supplementary role in reducing locomotor pressure and preventing slipping on muddy surfaces^26^. The expansion of the habitats of dinosaurs from forests to alluvial basins, including lakes and ponds, during the late Mesozoic as interpreted from the temporal limitation of dinosaur egg preservation in alluvial deposits to the late Mesozoic^27^ could have been related to the adaptation of polygonal skin texture on the foot pads of dinosaurs.

## Conclusions

**Figure 5:**
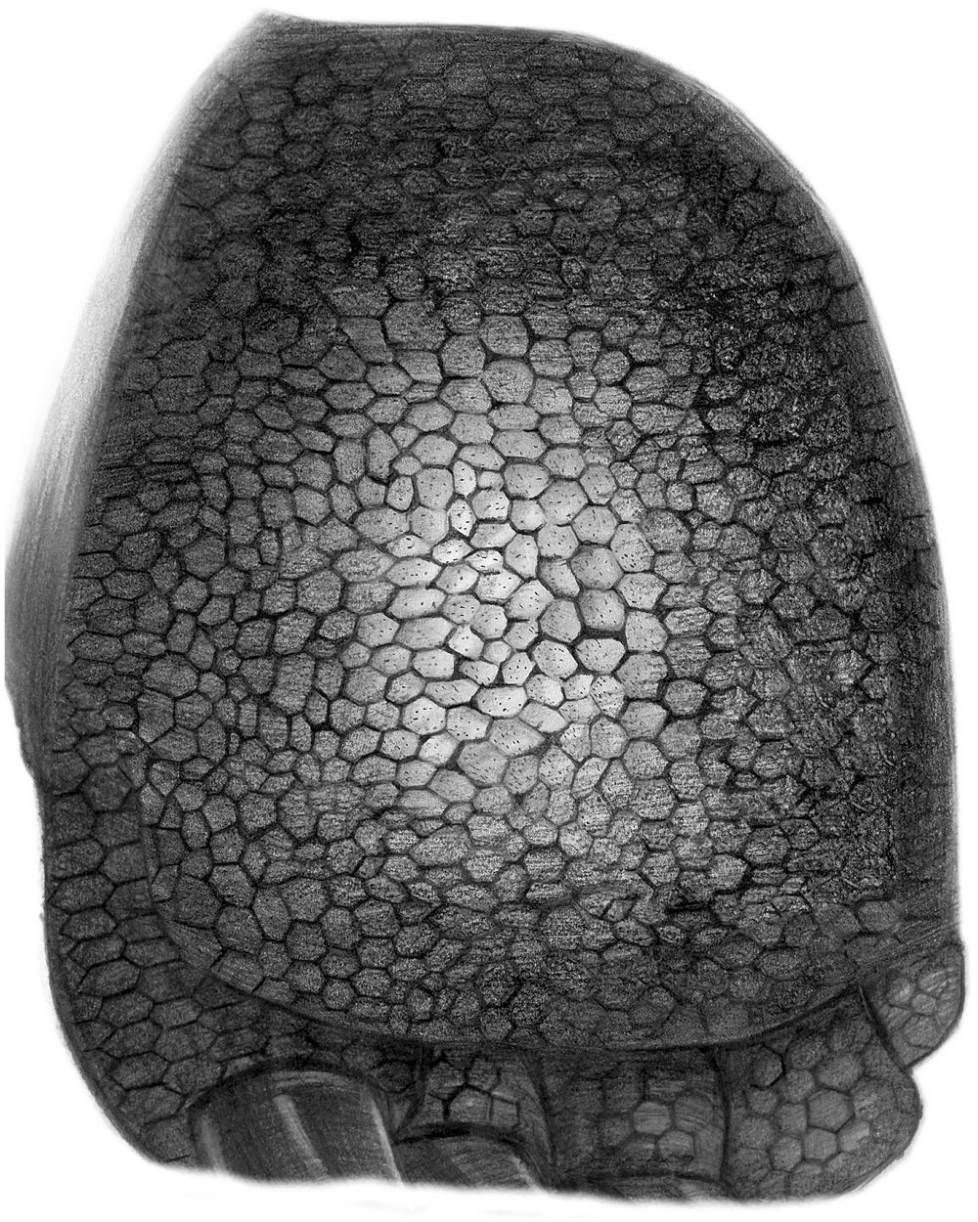
Reconstruction of the plantar surface of a sauropod foot with polygonal skin fabric (Drawn by Hyun Jeong Yoo).

The distinct skin impression and its cast observed in this study are the largest reported to date for a sauropod footprint. The interlaminated to interbedded nature of the sand with microbially active mud drapes a few millimetres thick under semi-arid paleoclimatic conditions could have enabled the polygonal skin texture to be impressed distinctly in the footprints. The observations here suggest that some sauropod dinosaurs in the Cretaceous had a well-developed polygonal skin texture covering nearly the whole of their foot pads to enhance traction (Fig. 5), as seen in the plantar surface with irregular networks of furrows of a modern elephant, and that dinosaurs would have adapted polygonal skin texture on their foot pads as they expanded their habitat into alluvial environments during the late Mesozoic.

## Material and Methods

In the field, the skin impression-bearing deposits were measured and photographed. The rock samples for sedimentological and taphonomic analysis were cut with a rock saw and polished. Thin sections were also made and were studied using an optical microscope (Nikon Optiphot2-POL). Photomicrographs were made with a Nikon Coolpix P50 for the thin sections and a Nikon Coolpix P300 and Samsung NX300 for the rocks. A ZEISS SUPRA™25 (FE-SEM, CARL ZEISS, Oberkochen, Germany) equipped with an Oxford X-max 80-mm^2^ Energy Dispersive Spectrometer (OXFORD INSTRUMENTS, Abingdon, Oxfordshire, UK) installed at PNU Core Research Institute was used for SEM analyses of the skin impression-bearing layer. All the samples were Pt-coated for SEM analysis. The operating conditions were 10 keV for SEM imaging and 15 keV for 30 live seconds for the EDS analyses. All the specimens are stored at the Department of Earth and Environmental Sciences of Pukyong National University, Busan, Republic of Korea.

## Electronic supplementary material

Supplementary information
