# Supplementary material for: A large and distinct skin impression on the cast of a sauropod dinosaur footprint from Early Cretaceous floodplain deposits, Korea
Source: Sci Rep. 2017 Nov 27;7:16339. doi: 10.1038/s41598-017-16576-y (PMC5703924; doi:10.1038/s41598-017-16576-y)
Supplement: Supplementary file 1 — Supplementary information [file 41598_2017_16576_MOESM1_ESM.doc]

**Supplementary information for:**

# A large and distinct skin impression on the cast of a sauropod dinosaur footprint from the Early Cretaceous floodplain deposits, Korea

In Sung Paik^1*^, Hyun Joo Kim^1^, Hoil Lee^1, 2^, & Seongyeong Kim^3^

1. Department of Earth and Environmental Sciences, Pukyong National University, Busan 48513, Republic of Korea ([paikis@pknu.ac.kr](mailto:paikis@pknu.ac.kr)).
2. Korea Institute of Geoscience and Mineral Resources, Daejeon 34132. Republic of Korea
3. School of Earth and Environmental Sciences, Seoul National University, Seoul 08826, Republic of Korea

Contents

1. Table S1
2. Supplementary Fig. s1
3. Supplementary Fig. s2
4. References
5. Table S1. List of skin impression reports in dinosaur footprints.

| **Geological Age** | **Formation** | **Country** | **Dinosaur** | **Remarks** |
| --- | --- | --- | --- | --- |
| Late Triassic | Fleming Fjord  Formation^S1^ | Greenland | Theropods | Patch  Slide mark (Scale scratch mark) |
| Middle Jurassic | Cloughton Formation^S2^  Scalby Formation^S2^ | UK | Sauropod | Patch  Slide mark (Scale scratch mark) |
| Late Jurassic | Lourinhã Formationa^S3^ | Portugal | Sauropod | Patch  Slide mark |
| Late Jurassic | Morrison Formation^S4^ | USA | Sauropod | - |
| Late Jurassic | Morrison Formation^S5^ | USA | Sauropod | Patch  Slide mark (Scale scratch mark) |
| Cretaceous  (Barremian) | Camaillas Formation^S6^ | Spain | Sauropod | Patch  Slide mark (Scale scratch mark) |
| Cretaceous  (Barremian) | Mirambel Formation^S7^ | Spain | Sauropod | Patch  Slide mark (Scale scratch mark) |
| Cretaceous | Ashdown Formation^S8^ | England | Ornithopod  (Iguanadont) | Patch  Slide mark |
| Cretaceous | Haman Formation^S9^ | S. Korea | Sauropod | Patch |
| Cretaceous (Albian –  Cenomanian)  Cretaceous (Cenomanian) | Chandler Formation^S10^  Dunvegan Formation^S10^ | USA  Canada | Ornithopod  (Ankylosaur) | Patch |
| Late Cretaceous | Cantwell Formation^S11^ | USA | Ornithopod  (Hadrosaur) | Patch |
| Late Cretaceous | Nemegt Formation^S12^ | Mongolia | Sauropod  Theropod | Patch  Slide mark (Scale scratch mark) |
| Late Cretaceous | Lance Formation^S13^ | USA | Ornithopod  (Hadrosaur) | Patch |

1. Supplementary Fig. s1.


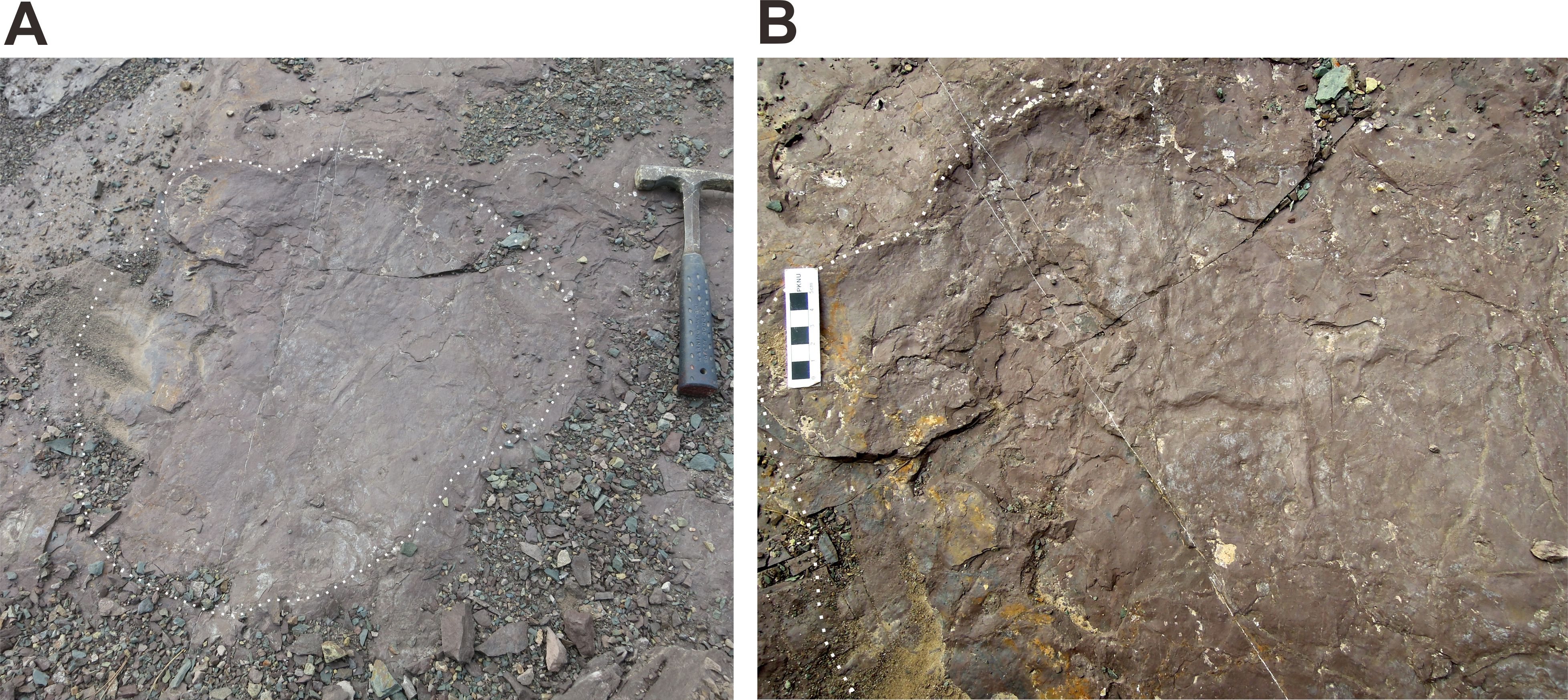


Supplementary Fig. s1. Diffuse skin impressions in patches on a dinosaur footprint observed in the examined deposits (A) and their close view (B).

1. Supplementary Fig. s2.


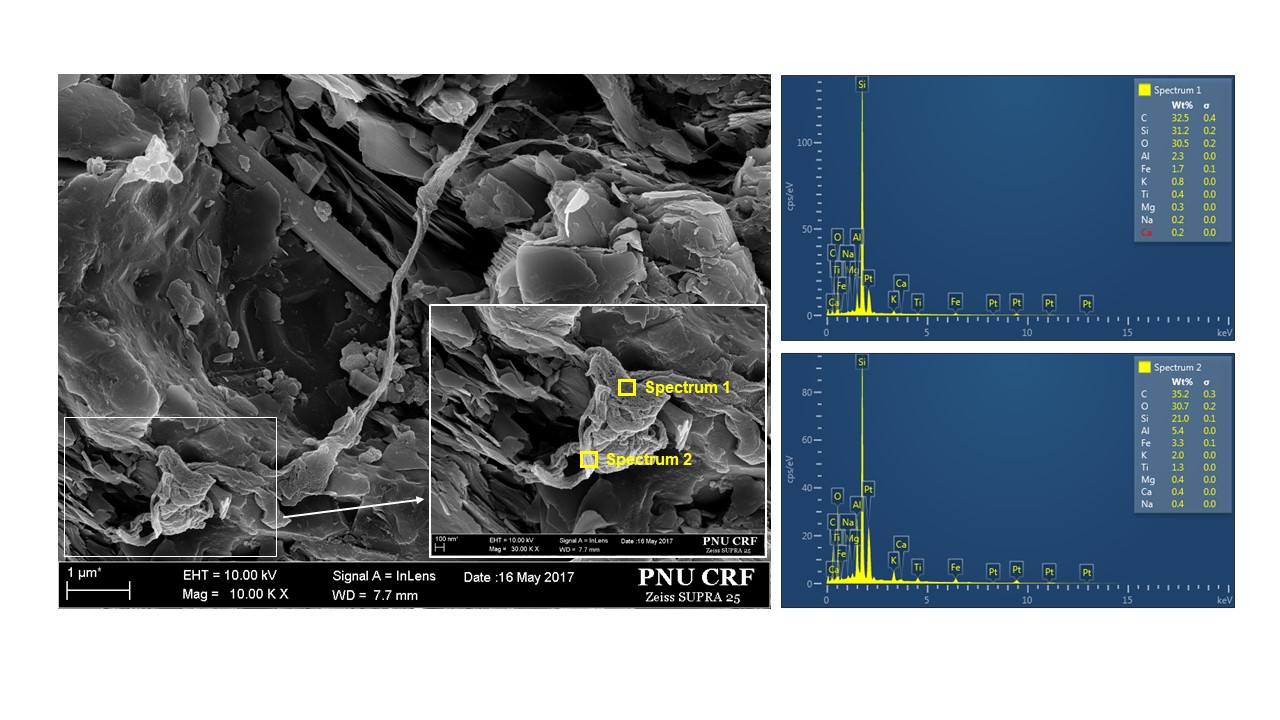


Supplementary Figure s2. EDS analysis for microbe showing the presence of organic matter in the distinct skin impression-bearing mudstone.

1. References

S1. Gatesy, S. M. Skin impressions of Triassic theropods as records of foot movement. *Bull. Mus. Comp. Zool*. **156**, 137-149 (2001)

S2. Mateus, O. & Milàn, J. A diverse Upper Jurassic dinosaur ichnofauna from central-west Portugal. *Lethaia* **43**, 245-257 (2010)

S3. Romano, M. & Whyte, M. A. Information on the foot morphology, pedal skin texture and limb dynamics of sauropods: evidence from the ichnological record of the Middle Jurassic of the Cleveland Basin, Yorkshire, UK. *Zubía* **30**, 45-92 (2012)

S4. Lockley, M. G. & Hunt, A. P. *Dinosaur Tracks and Other Fossil Footprints of the Western United States* (Columbia Univ. Press, 1995)

S5. Platt, B. F. & Hasiotis, S. T. Newly discovered sauropod dinosaur tracks with skin and foot-pad impressions from the Upper Jurassic Morrison Formation, Bighorn Basin, Wyoming, U.S.A. *Palaios* **21**, 249-261 (2006)

S6. Navarrete, R., *et al*. A thick Tethyan multi-bed tsunami deposit preserving a dinosaur megatracksite within a coastal lagoon (Barremian, eastern Spain). *Sed. Geol.* **313**, 105-127 (2014)

S7. Castanera, D. *et al*. An overview of the Lower Cretaceous dinosaur tracksites from the Mirabel Formation in the Iberian Range (NE Spain). *New Mexico Mus. Nat. His. Sci. Bull.* **71**, 65-74 (2016)

S8. Davies, N. S., Liu, A. G., Gibling, M. R. & Miller, R. F. Resolving MISS conceptions and misconceptions: A geological approach to sedimentary surface textures generated by microbial and abiotic processes*. Earth-Sci. Rev.* **154**, 210-246 (2016)

S9. Lockley, M.G. *et al*. Dinosaur-dominated footprint assemblages from the Cretaceous Jindong Formation, Hallyo Haesang National Park area, Goseong Country, South Korea: evidence and applications. *Cret. Res.* **27**, 70–101 (2006)

S10. McCrea, R., Lockley, M. G. & Meyer, C. A. in *The Armored Dinosaurs* (ed Carpenter, K.) 413-454 (Indiana Univ. Press, 2001)

S11. Fiorillo, A. R., Hasiotis, S. T. & Kobayashi, Y. Herd structure in Late Cretaceous polar dinosaurs: a remarkable new dinosaur tracksite, Denali National Park, Alaska, USA. *Geology* **42**, 719-722 (2014)

S12. Currie, P. J., Badamgarav, D. & Koppelhus, E. B. The first Late Cretaceous footprints from the Nemegt Locality in the Gobi of Mongolia. *Ichnos* **10**, 1-13 (2003)

S13. Lockley, M. G., Nadon, G. & Currie, P. J. A diverse dinosaur-bird footprint assemblage from the Lance Formation, Upper Cretaceous, Eastern Wyoming: implications for ichnotaxonomy. *Ichnos* **11**, 229-249 (2003)
